# Evaluation of cardiovascular ischemic event rates in dasatinib-treated patients using standardized incidence ratios

**DOI:** 10.1007/s00277-017-3012-z

**Published:** 2017-05-22

**Authors:** Giuseppe Saglio, Philipp le Coutre, Jorge Cortes, Jiří Mayer, Philip Rowlings, François-Xavier Mahon, Glenn Kroog, Kyna Gooden, Milayna Subar, Neil P. Shah

**Affiliations:** 1 Clinical and Biological Sciences of the University of Turin, San Luigi Hospital, 10043 Orbassano-Torino, Italy; 2 Charité, Campus Virchow Klinikum, Universitätsmedizin Berlin, Berlin, Germany; 3 The University of Texas MD Anderson Cancer Center, Houston, TX USA; 4 Department of Internal Medicine, Hematology and Oncology, Masaryk University Hospital Brno, Brno, Czech Republic; 5 Calvary Mater Newcastle Hospital, University of Newcastle, Waratah, NSW Australia; 6 Laboratoire d’Hématologie et Service des Maladies du Sang, Bordeaux et Institut Bergonié, Centre Hospitalier Universitaire de Bordeaux, Bordeaux, France; 7 Bristol-Myers Squibb, Princeton, NJ USA; 8 UCSF School of Medicine, San Francisco, CA USA

**Keywords:** Chronic myeloid leukemia, Tyrosine kinase inhibitors, Dasatinib, Cardiovascular, Ischemic

## Abstract

With high survival rates for chronic myeloid leukemia (CML) patients treated with BCR-ABL1 tyrosine kinase inhibitors (TKIs), emerging consequences, such as arterial ischemic events, require consideration when evaluating treatment options. Cardiovascular ischemic event incidence in clinical trials was evaluated in 2712 dasatinib-treated patients with Philadelphia chromosome-positive (Ph+) leukemias from 11 first- and second-line trials (pooled), newly diagnosed CML patients treated with dasatinib or imatinib (DASISION), and prostate cancer patients treated with dasatinib or placebo plus docetaxel/prednisone (READY). Overall, 2–4% of dasatinib-treated patients had cardiovascular ischemic events. Most dasatinib-treated patients with an event had a history of and/or risk factor for atherosclerosis (pooled 77 with history/risk and event/96 with events; DASISION 8/10; READY 15/18). Most cardiovascular ischemic events occurred within 1 year of initiating dasatinib (pooled 69/96; DASISION 7/10; READY 16/18). Comparison of observed and expected event rates through standardized incidence ratios indicates that dasatinib does not increase risk for cardiovascular ischemic events compared with external reference populations.

**Electronic supplementary material:**

The online version of this article (doi:10.1007/s00277-017-3012-z) contains supplementary material, which is available to authorized users.

## Introduction

Treatment of chronic phase chronic myeloid leukemia (CML-CP) with a BCR-ABL1-targeted tyrosine kinase inhibitor (TKI) has transformed a once fatal disease into one that is controlled in >90% of patients [1]. As most patients need to continue lifelong therapy to maintain disease control, long-term safety is becoming increasingly important to identify potential new adverse events (AEs) that may arise during extended treatment.

Arterial ischemic events have emerged as AEs associated with TKI therapy. Currently, four of the five BCR-ABL1 TKIs approved for the treatment of CML (imatinib, dasatinib, nilotinib, and ponatinib) have one or more AEs related to arterial ischemia reported in the prescribing information; however, the incidence of arterial ischemic events is lower with imatinib and dasatinib compared with the incidence with nilotinib and ponatinib [2–5]. Ponatinib was temporarily withdrawn from the US market in 2014 due to patients experiencing high rates of serious vascular AEs [6, 7], bringing risk assessment of cardiovascular events to the forefront for patients prescribed BCR-ABL1 TKIs.

The type and severity of ischemic events vary across the TKIs. Dasatinib has been associated with pulmonary arterial hypertension (PAH) [4], but the mechanism and any factors potentially related to development of PAH are unknown. Dasatinib should be discontinued in patients with documented PAH, which then typically leads to partial or complete reversal of the condition [4]. A strong association between nilotinib and multiple vascular AEs (femoral artery stenosis, coronary artery stenosis, intermittent claudication, and peripheral arterial disease [PAD]) was identified through an analysis of data from the FDA Adverse Event Reporting System (FAERS) database [8]. Also, in the 5-year report of the Evaluating Nilotinib Efficacy and Safety in Clinical Trials–Newly Diagnosed Patients (ENESTnd) study, 8 and 13% of patients who received 300 or 400 mg nilotinib, respectively, experienced some form of cardiovascular event, including ischemic heart disease, an ischemic cerebrovascular event, or PAD [9]. In patients with CML, phase I and II clinical trials investigating the use of ponatinib reported serious vascular events in approximately 48 and 24% of patients, respectively [6, 7].

Recently, two separate groups have performed a meta-analysis investigating the potential association of the currently available BCR-ABL1 TKIs with vascular AEs. Findings from Chai et al. concluded that patients who received nilotinib or ponatinib experienced a greater number of major arterial events than patients who received imatinib, dasatinib, or non-TKI-based treatment [10]. Douxfils et al. analyzed the incidence of vascular occlusive events in patients with CML taking one of the five approved BCR-ABL1 TKIs. Their overall conclusions were that there is an increased risk of vascular occlusive events with all new-generation TKIs compared with imatinib [11]. Retrospective meta-analysis, however, is limited in its ability to draw conclusions due to potential inconsistency among studies in definitions of vascular AEs as well as variations in study selection methods, duration of patient follow-up across datasets, and inclusion and exclusion criteria. The analysis presented here will focus on a broader set of clinical trial data within the dasatinib trial program to gain a better and deeper understanding of cardiovascular event rates in dasatinib-treated patients.

After a 5-year follow-up of the phase III DASatinib versus Imatinib Study In treatment-Naïve CML patients (DASISION) study, 4% of dasatinib-treated patients experienced a cardiovascular ischemic event compared with 2% of imatinib-treated patients [12]. Therefore, we evaluated the occurrence of cardiovascular ischemic events across the dasatinib clinical trial program. Cardiovascular ischemic events were assessed for dasatinib-treated patients with Philadelphia chromosome-positive (Ph+) leukemias in a pooled population of 11 trials and for patients in the dasatinib and non-dasatinib comparator arms from both DASISION and a phase III trial in patients with prostate cancer (READY). To determine whether the frequency of these events in clinical trials is similar to the expected incidence in similar populations from the community setting, the expected rate of occurrence of cardiovascular ischemic events was estimated in external reference populations generated from an insurance claims database. Using standardized incidence ratios (SIRs), these estimates were then compared with the rate of occurrence of cardiovascular ischemic events in the dasatinib clinical trial program.

## Methods

### Patient populations

Three dasatinib clinical trial populations were investigated (Table 1): a large, pooled population of Ph+ dasatinib-treated patients, including those from DASISION; dasatinib- and imatinib-treated patients from the randomized phase III DASISION trial [13]; and patients treated with dasatinib or placebo plus docetaxel/prednisone from the randomized phase III READY prostate cancer trial [14] (see Online Resource Table S1).

**Table 1:** Dasatinib clinical trial and external reference populations

		Men (%)	Median age (years)	Median duration of therapy (months (range))
Pooled Ph+ population (*N* = 2712)	Dasatinib 100–140 mg daily	53	54	19.2 (0–93.2)
DASISION (*N* = 519)	Dasatinib 100 mg QD (*n* = 259)	56	46	60.5 (<0.1–72.7)
	Imatinib 400 mg QD (*n* = 260)	63	49	60.3 (0.3–74.6)
READY (*N* = 1522)	Dasatinib 100 mg QD + docetaxel/prednisone (*n* = 762)	100	69	8.1 (0.1–41.4)
	Placebo + docetaxel/prednisone (*n* = 760)	100	68	8.4 (<0.1–41.3)
External disease-based populations (US claims database)	CML (*N* = 16,000)	56	59	–
	Prostate cancer (*N* = 530,000)	100	66	–
External general populations (US claims database)	General (*N* = 116,000,000)	48	42	–
	General male (*N* = 56,000,000)	100	42	–

*CML* chronic myeloid leukemia, *Ph+* Philadelphia chromosome-positive, *QD* once daily

Eligibility criteria and patient characteristics have been described [12–23]. Patients in the pooled Ph+ population had CML-CP (*n* = 1618) or advanced disease (CML in accelerated/blast phase [CML-AP/BP] or Ph+ acute lymphoblastic leukemia [Ph+ ALL]; *n* = 1094). They were treated with first- or second-line dasatinib 15 to 240 mg daily in one of 11 phase I/II/III clinical trials. All procedures followed were in accordance with the ethical standards of the responsible committee on human experimentation (institutional and national) and with the Helsinki Declaration of 1975, as revised in 2000–2008. Informed consent was obtained from all patients for being included in the study.

Data from a large, population-based US health insurance claims database, MarketScan® Commercial Claims and Medicare Supplemental (Truven Health Analytics, Ann Arbor, MI, USA), from 2008 to 2013, were used to generate the external reference populations (see Online Resource Methods). Inclusion dates were based on available licensed data. The external reference populations included disease-based populations of patients with CML and prostate cancer, as well as general populations of all eligible patients and men only (Table 1).

### Analyses

Cardiovascular ischemic events evaluated in the dasatinib clinical trials were identified using preferred terms from the Medical Dictionary for Regulatory Activities (MedDRA) [24] (see Online Resource Methods). Preexisting risk of cardiovascular ischemic events was evaluated in the clinical trial patient subsets with and without a cardiovascular ischemic event on the basis of risk factors provided at enrollment, including factors listed in Online Resource Methods.

Follow-up in all external reference populations occurred in parallel with trial populations. Cases of cardiovascular ischemic events were defined by at least two instances of a MedDRA preferred term code during the follow-up period. Patients with the code identified before the follow-up period were excluded from analysis in an attempt to mimic entry criteria for the dasatinib clinical trials.

SIRs were calculated to evaluate observed incidence rates of cardiovascular ischemic events in dasatinib clinical trials compared with the expected incidence based on rates in an external reference population. Expected rates were calculated for each age and sex stratum and were summed to provide an overall expected incidence rate in the dasatinib-treated population. The observed rate in dasatinib-treated patients in the clinical trial populations was then divided by the expected rate in the appropriate reference population to determine the SIR and a 95% confidence interval (CI). Calculations were performed for the dasatinib clinical trials compared with both appropriate external disease-based and general populations. Specifically, patients from the pooled Ph+ population (all 2712 patients with CML-CP, CML-AP/BP, and Ph+ ALL) and DASISION were compared with the general and CML external reference populations, and patients from READY were compared with the general male and prostate cancer external reference populations.

## Results

### Incidence and timing of cardiovascular ischemic events in dasatinib clinical trials

Characteristics of patients from dasatinib clinical trials and the external reference populations are described in Table 1. There were similar numbers of men and women in the clinical trials and the external general and CML reference populations. Median age was similar in READY and the external prostate cancer reference population, but these were both higher compared with the other clinical trial and external reference populations. The external general population had the lowest median age. Median duration of dasatinib therapy was 19 months for the pooled Ph+ population (29.0 months for CML-CP and 6.2 months for CML-AP/BP/Ph+ ALL), 60 months for both arms of DASISION, and 8 months for both arms of READY.

In all three dasatinib clinical trial populations, the incidence of any cardiovascular ischemic event (defined in Online Resource Methods) ranged from 2 to 4% (Table 2). In the pooled Ph+ population, 55 (2%) patients had cardiovascular ischemic events that were grade 3/4, and five (<1%) patients died due to a cardiovascular ischemic event. Dasatinib dose and CML disease phase do not appear to be associated with increased risk of these events in the pooled Ph+ population because event incidence was similar for patients with chronic or advanced CML at different doses of dasatinib. In DASISION, a total of 10 dasatinib-treated patients (4%) experienced cardiovascular ischemic events compared with four patients (2%) who received imatinib (Table 2). Of the reported events, five were grade 3/4 with dasatinib and two were grade 3/4 with imatinib. In READY, 18 patients (2%) receiving dasatinib experienced cardiovascular ischemic events compared with nine patients (1%) given placebo (Table 2). Ten cardiovascular ischemic events with dasatinib were grade 3/4 and six events with placebo were grade 3/4. The most distinguishing event between arms was myocardial infarction, where seven (1%) and one (<1%) patients experienced this event in the dasatinib and placebo arms, respectively.

**Table 2:** Cardiovascular ischemic events

	Dasatinib-treated patients from pooled Ph+ population, *n* (%)					
	CML-CP		CML-AP/BP or Ph+ ALL			Ph+ leukemias (*N* = 2712)
	100 mg QD (*n* = 548)	All (*n* = 1618)	140 mg QD (*n* = 304)		All (*n* = 1094)	
Any CV ischemic event^a^	17 (3.10)	65 (4.02)	6 (1.97)		31 (2.83)	96 (3.54)
MI	9 (1.64)	21 (1.30)	2 (0.66)		12 (1.10)	33 (1.22)
Angina pectoris	5 (0.91)	31 (1.92)	2 (0.66)		14 (1.28)	45 (1.66)
CAD	3 (0.55)	11 (0.68)	0		0	11 (0.41)
Other^b^	1 (0.18)	10 (0.62)	3 (0.99)		10 (0.91)	20 (0.74)
	Treated patients from DASISION, *n* (%)					
	Dasatinib 100 mg QD (*n* = 258)			Imatinib 400 mg QD (*n* = 258)		
	Any grade	Grade 3/4	Grade 5	Any grade	Grade 3/4	Grade 5
Any CV ischemic event^a^	10 (3.88)	5 (1.94)	2 (0.78)	4 (1.55)	2 (0.78)	1 (0.39)
MI	6 (2.33)	4 (1.55)	2 (0.78)	2 (0.78)	1 (0.39)	1 (0.39)
Angina pectoris	3 (1.16)	1 (0.39)	0	2 (0.78)	0	0
CAD	1 (0.39)	0	0	0	0	0
Acute coronary syndrome	0	0	0	1 (0.39)	1 (0.39)	0
	Treated patients from READY, *n* (%)					
	Dasatinib 100 mg QD + docetaxel/prednisone (*n* = 761)		Placebo + docetaxel/prednisone (*n* = 757)			
	Any grade	Grade 3/4	Any grade			Grade 3/4
Any CV ischemic event^a^	18 (2.37)	10 (1.31)	9 (1.19)			6 (0.79)
MI	7 (0.92)	6 (0.79)	1 (0.13)			1 (0.13)
Angina pectoris	7 (0.92)	1 (0.13)	6 (0.79)			3 (0.40)
Myocardial ischemia	3 (0.39)	1 (0.13)	3 (0.40)			2 (0.26)
Troponin increased^c^	2 (0.26)	1 (0.13)	1 (0.13)			0
CAD	1 (0.13)	0	1 (0.13)			1 (0.13)
Acute coronary syndrome	1 (0.13)	1 (0.13)	1 (0.13)			1 (0.13)
Coronary artery occlusion	1 (0.13)	1 (0.13)	0			0

*ALL* acute lymphoblastic leukemia, *AP/BP* accelerated/blast phase, *CAD* coronary artery disease, *CML* chronic myeloid leukemia, *CP* chronic phase, *CV* cardiovascular, *MI* myocardial infarction, *Ph+* Philadelphia chromosome-positive, *QD* once daily

^a^Patients may have had more than one event within a class

^b^Includes acute coronary syndrome, electrocardiogram T-wave abnormal, troponin I, troponin I increased, troponin increased, troponin T, and troponin T increased

^c^Includes troponin I increased and troponin T increased

Any history of and/or risk factors for atherosclerosis were also taken into account in this assessment of cardiovascular ischemic events. In the pooled Ph+ population, 47% of patients had a prior history of and/or had risk factors for atherosclerosis (Table 3). The particular preexisting conditions and risk factors identified are detailed in Table 3. Of the 96 cardiovascular ischemic events in the pooled Ph+ population, 77 (80%) were reported in these patients with a history of and/or risk factors for atherosclerosis. The incidence of cardiovascular ischemic events in the population with known risk factors was 6% compared with 1% in those without reported risk factors. In DASISION, 40 and 46% of patients had a history of and/or risk factors for atherosclerosis in the dasatinib and imatinib arms, respectively. The majority of events in both arms were reported among patients with a history and/or risk factors for atherosclerosis: eight of 10 (80%) cardiovascular ischemic events with dasatinib and three of four (75%) with imatinib. A majority of patients from READY, 66% of patients on dasatinib and 64% of patients on placebo, had a history of and/or risk factors for atherosclerosis (Table 3). Of the 18 dasatinib-treated patients with a cardiovascular ischemic event in the READY trial, 15 (83%) had a history of and/or risk factors for atherosclerosis along with six of nine (67%) patients in the placebo population.

**Table 3:** Baseline history of and/or risk factors for atherosclerosis

	Treated patients, *n* (%)				
	Pooled Ph+ population (*N* = 2712)	DASISION		READY	
		Dasatinib 100 mg QD (*n* = 258)	Imatinib 400 mg QD (*n* = 258)	Dasatinib 100 mg QD + docetaxel/prednisone (*n* = 761)	Placebo + docetaxel/prednisone (*n* = 757)
History of and/or risk factor for atherosclerosis^a^					
Yes	1280 (47.20)	104 (40.31)	119 (46.12)	504 (66.23)	483 (63.80)
CV ischemic events	77 (6.02)	8 (7.69)	3 (2.52)	15 (2.98)	6 (1.24)
No CV ischemic events	1203 (93.98)	96 (92.31)	116 (97.48)	489 (97.02)	477 (98.76)
No	1432 (52.80)	154 (59.69)	139 (53.88)	257 (33.77)	274 (36.20)
CV ischemic events	19 (1.33)	2 (1.30)	1 (0.72)	3 (1.17)	3 (1.09)
No CV ischemic events	1413 (98.67)	152 (98.70)	138 (99.28)	254 (98.83)	271 (98.91)
History of atherosclerosis					
Preexisting IHD	165 (6.08)	9 (3.49)	13 (5.04)	106 (13.93)	98 (12.95)
Preexisting non-cardiac atherosclerosis	104 (3.83)	10 (3.88)	10 (3.88)	44 (5.78)	48 (6.34)
Risk factors for atherosclerosis^b^					
Current smoker	242 (8.92)	31 (12.02)	50 (19.38)	39 (5.12)	36 (4.76)
Former smoker	401 (14.79)	23 (8.91)	20 (7.75)	50 (6.57)	55 (7.27)
Hypertension	625 (23.05)	47 (18.22)	43 (16.67)	370 (48.62)	346 (45.71)
Diabetes	245 (9.03)	19 (7.36)	13 (5.04)	116 (15.24)	131 (17.31)
Hypercholesterolemia	216 (7.96)	25 (9.69)	23 (8.91)	168 (22.08)	161 (21.27)

*CV* cardiovascular, *IHD* ischemic heart disease, *Ph+* Philadelphia chromosome-positive, *QD* once daily

^a^Patients may have had both a history of and risk factors for atherosclerosis

^b^Patients may have had more than one risk factor

The incidence of cardiovascular ischemic events increased with age in the patients with CML in the pooled Ph+ population and in DASISION. Of those aged ≤44 years, 1 and 2% from the pooled Ph+ population and DASISION, respectively, experienced an event compared with ≥10% in patients aged ≥75 years (Table 4). No increase in cardiovascular ischemic events with age was observed in patients from the READY study.

**Table 4:** Cardiovascular ischemic events by age

	Dasatinib-treated patients from the pooled Ph+ population, *n* (%)					
	Total	≤44 years	45–64 years	65–74 years	≥75 years	
Total patients	2712 (100)	835 (30.79)	1260 (46.46)	494 (18.22)	123 (4.54)	
CV ischemic event	96 (3.54)	9 (1.08)	44 (3.49)	31 (6.28)	12 (9.76)	
No CV ischemic event	2616 (96.46)	826 (98.92)	1216 (96.51)	463 (93.72)	111 (90.24)	
	Treated patients from DASISION, *n* (%)					
	Dasatinib 100 mg QD					Imatinib 400 mg QD
	Total	≤44 years	45–64 years	65–74 years	≥75 years	Total
Total patients	258 (100)	120 (46.51)	113 (43.80)	18 (6.98)	7 (2.71)	258 (100)
CV ischemic event	10 (3.88)	2 (1.67)	5 (4.42)	1 (5.56)	2 (28.57)	4 (1.55)
No CV ischemic event	248 (96.12)	118 (98.33)	108 (95.58)	17 (94.44)	5 (71.43)	254 (98.45)
	Treated patients from READY, *n* (%)					
	Dasatinib 100 mg QD + docetaxel/prednisone					Placebo + docetaxel/prednisone
	Total	≤44 years	45–64 years	65–74 years	≥75 years	Total
Total patients	761 (100)	N/A	251 (32.98)	333 (43.76)	177 (23.26)	757 (100)
CV ischemic event	18 (2.37)	N/A	4 (1.59)	10 (3.00)	4 (2.26)	9 (1.19)
No CV ischemic event	743 (97.63)	N/A	247 (98.41)	323 (97.00)	173 (97.74)	748 (98.81)

*CV* cardiovascular, *N/A* not applicable, *Ph+* Philadelphia chromosome-positive, *QD* once daily

Most of the cardiovascular ischemic events in all three clinical trial populations occurred within the first year of initiating dasatinib, the majority within the first 6 months. In the pooled Ph+ population, 69 of 96 events (72%) occurred during the first year of dasatinib therapy (Table 5), with 57 occurring in the first 6 months and only 27 after 1 year. Seven of 10 events (70%) from DASISION and 16 of 18 events (89%) from READY also occurred during the first year of treatment. After the first 6 months of treatment, the incidence of cardiovascular ischemic events was similar for the dasatinib and comparator arms in both trials, and overall, there was no increase in ischemic events with exposure to dasatinib over time. The incidence in at-risk patients was calculated after the first year of treatment, taking into account those patients who had already experienced an on-study cardiovascular ischemic event (Table 5). Incidence for the pooled Ph+ population and in DASISION was 3% in the first year and decreased to 0 to 1% in at-risk populations in subsequent years.

**Table 5:** Time to first cardiovascular event by age

Timing of first CV ischemic event	Dasatinib-treated patients from pooled Ph+ population, *n* (%)						
	Total (*N* = 2712)	≤44 years (*n* = 835)	45–64 years (*n* = 1260)	65–74 years (*n* = 494)	≥75 years (*n* = 123)	Patients with event/at risk	
0 to <6 months	57 (2.10)	6 (0.72)	25 (1.98)	20 (4.05)	6 (4.88)	69/2712 (2.54)	
6 to <12 months	12 (0.44)	0	6 (0.48)	4 (0.81)	2 (1.63)		
1 to <2 years	11 (0.41)	1 (0.12)	5 (0.40)	4 (0.81)	1 (0.81)	11/1617 (0.68)	
2 to <3 years	5 (0.18)	1 (0.12)	3 (0.24)	0	1 (0.81)	5/1202 (0.42)	
3 to <4 years	5 (0.18)	0	3 (0.24)	2 (0.40)	0	5/847 (0.59)	
4 to <7 years	6 (0.22)	1 (0.12)	2 (0.16)	1 (0.20)	2 (1.63)	6/696 (0.86)	
	Treated patients from DASISION, *n* (%)						
	Dasatinib 100 mg QD						Imatinib 400 mg QD
Timing of first CV ischemic event	Total (*N* = 258)	≤44 years (*n* = 120)	45–64 years (*n* = 113)	65–74 years (*n* = 18)	≥75 years (*n* = 7)	Patients with event/at risk	Total (*N* = 258)
0 to <6 months	4 (1.55)	1 (0.83)	2 (1.77)	1 (5.56)	0	7/258 (2.71)	0
6 to <12 months	3 (1.16)	0	2 (1.77)	0	1 (14.29)		1 (0.39)
1 to <2 years	0	0	0	0	0	0/227	0
2 to <3 years	2 (0.78)	0	1 (0.88)	0	1 (14.29)	2/207 (0.97)	2 (0.78)
3 to <6 years	1 (0.39)	1 (0.83)	0	0	0	1/185 (0.54)	1 (0.39)
	Treated patients from READY, *n* (%)						
	Dasatinib 100 mg QD + docetaxel/prednisone						Placebo + docetaxel/prednisone
Timing of first CV ischemic event	Total (*N* = 761)	≤44 years	45–64 years (*n* = 251)	65–74 years (*n* = 333)	≥75 years (*n* = 177)	Patients with event/at risk	Total (*N* = 757)
0 to <6 months	14 (1.84)	N/A	1 (0.40)	9 (2.70)	4 (2.26)	16/761 (2.10)	7 (0.92)
6 to <12 months	2 (0.26)	N/A	1 (0.40)	1 (0.30)	0		2 (0.26)
1 to <2 years	2 (0.26)	N/A	2 (0.80)	0	0	2/255 (0.78)	0
2 to <6 years	0	N/A	0	0	0	0	0

*CV* cardiovascular, *N/A* not applicable, *Ph+* Philadelphia chromosome-positive, *QD* once daily

Due to the varying follow-up times and numbers of patients enrolled across these trials, incidence rates of cardiovascular ischemic events based on patient-years of dasatinib exposure were determined. Incidence in the pooled population was 1.5 events/100 patient-years. Rates were 0.9 and 0.4 events/100 patient-years for the dasatinib and imatinib arms, respectively, in DASISION. Dasatinib- and placebo-treated patients from the READY trial were calculated to have had 3.0 and 1.4 events/100 patient-years, respectively.

### Standardized incidence ratios

**Fig. 1:**
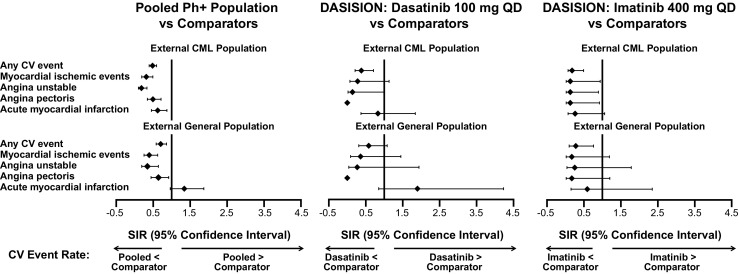
Standardized incidence ratios of cardiovascular ischemic event rates among patients from the pooled *Ph+* population and DASISION and external reference populations. Standardized incidence ratios (SIRs; *diamonds*) were calculated for the pooled *Ph+* population and patients in the dasatinib and imatinib arms of DASISION. Observed CV event values from the study analysis were compared to expected values in both an external CML population and a general population, and 95% confidence intervals (CIs; *horizontal bars*) were calculated. The *vertical line* denotes a SIR of 1.0. If a SIR 95% CI range includes the value of 1.0, it is suggested that the event occurred as expected. If the SIR 95% CI is <1.0, it is suggested that the event occurred less frequently than expected and if it is >1, more frequently than expected. Any CV event includes myocardial ischemic events (including electrocardiogram signs of myocardial ischemia), angina unstable (including acute coronary syndrome, coronary artery occlusion, and troponin I increased), angina pectoris, acute myocardial infarction (including troponin I, silent MI, and MI), arteriosclerosis coronary artery, coronary artery disease, troponin T, troponin T increased, electrocardiogram T-wave abnormal, and coronary arterial stent insertion. *CML* chronic myeloid leukemia, *CV* cardiovascular, *Ph+* Philadelphia chromosome-positive, *QD* once daily

**Fig. 2:**
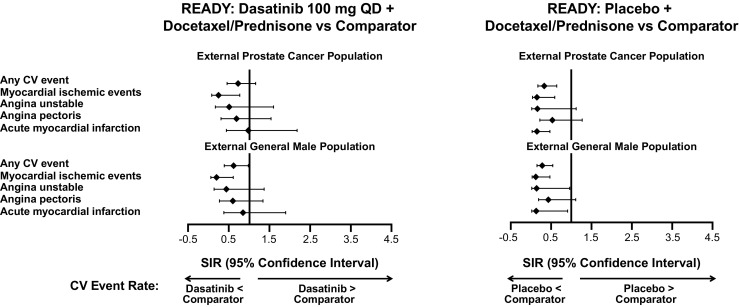
Standardized incidence ratios of cardiovascular ischemic event rates among patients from READY and external reference populations. Standardized incidence ratios (SIRs; *diamonds*) were calculated for patients in the dasatinib and placebo arms of the READY trial. Observed CV event values from the READY trial were compared to the expected number of events from both an external prostate cancer population and a general male population, and 95% confidence intervals (CIs; *horizontal bars*) were calculated. The *vertical line* denotes a SIR of 1.0. If a SIR 95% CI range includes the value of 1.0, it is suggested that the event occurred as expected. If the SIR 95% CI is <1, it is suggested that the event occurred less than expected and if it is >1, more than expected. Any CV event includes myocardial ischemic events (including electrocardiogram signs of myocardial ischemia), angina unstable (including acute coronary syndrome, coronary artery occlusion, and troponin I increased), angina pectoris, acute myocardial infarction (including troponin I, silent MI, and MI), arteriosclerosis coronary artery, coronary artery disease, troponin T, troponin T increased, electrocardiogram T- wave abnormal, and coronary arterial stent insertion. *CV* cardiovascular, *QD* once daily

In this analysis, a SIR 95% CI range that does not fall across a value of 1.0 indicates that the incidence of the event in clinical trials is statistically different than expected based on comparison to the reference population. Based on the SIRs, the overall rates of cardiovascular ischemic events observed in dasatinib-treated patients from the pooled Ph+ population were not higher than expected when compared with the external CML and general populations (Fig. 1). Acute myocardial infarction occurred at a higher rate than expected (SIR >1.0), but the differences relative to external reference populations were not statistically significant (95% CI included 1.0). Similarly, using SIRs, cardiovascular ischemic event rates in either treatment arm of DASISION were not higher than expected when compared with the external reference populations, except for acute myocardial infarction (Fig. 1). However, as in the pooled Ph+ population, this difference was not significant. All cardiovascular ischemic events were reported less often than expected in both dasatinib- and placebo-treated patients in the READY study (Fig. 2).

## Discussion

Arterial ischemic events have been reported in association with TKI use in patients with CML [2–5]. In this analysis, cardiovascular ischemic events were investigated in patients from dasatinib clinical trials that enrolled patients with Ph+ leukemia and prostate cancer and found that a low percentage (2–4%) experienced an event while receiving dasatinib. The highest cardiovascular ischemic event incidence rate per 100 patient-years observed in this analysis was for dasatinib-treated patients in the READY study (3.0/100 patient-years). This was higher than the rate for placebo-treated patients in the READY study (1.4/100 patient-years), dasatinib-treated patients in DASISION (0.9/100 patient-years), and patients in the pooled population treated with dasatinib (1.5/100 patient-years). Most cardiovascular events occurred in the first year of dasatinib treatment, supporting lack of a cumulative drug effect, although additional studies are required to help determine whether there is an acute process for the development of these events early after initiation of therapy.

SIRs analysis demonstrated that cardiovascular ischemic event rates in dasatinib-treated patients in clinical trials were not higher than would be expected relative to comparable external populations. This finding is consistent with the results from the FAERS analysis, which also did not show an association between dasatinib and cardiovascular ischemic events [8].

The list of cardiovascular ischemic events used in the analysis presented here did not include peripheral or cerebrovascular ischemic events. A retrospective analysis specifically examining reports of PAD/PAD-related events, which included the 11 clinical trials that composed the pooled Ph+ population here, reported a cumulative event incidence of 0.4% (11/2712) for dasatinib-treated patients [25]. Also, within the pooled Ph+ population, 30 of 2712 patients (1%) reported a cerebrovascular event. With this low incidence of PAD and cerebrovascular ischemic events, it is likely that inclusion of them in this analysis of cardiovascular ischemic event incidence would not alter the overall interpretation of the results.

The phase III DASISION and READY trials each had a comparator arm that did not receive dasatinib and was administered either imatinib or placebo, respectively. With the overall low incidence and the lack of a significant difference among treatment arms in comparative trials, we cannot conclude that dasatinib confers a greater risk for vascular ischemia.

There are some data indicating that nilotinib and ponatinib may confer a greater risk for vascular ischemic events than imatinib and dasatinib [2–5]; however, cross-trial comparisons are difficult due to varying reporting requirements, enrollment criteria, and the choice of terms used to define a cardiovascular event from one trial to the next [26]. For example, in DASISION, patients with a history of myocardial infarction were only excluded if an event had occurred within the previous 6 months [13], whereas all patients with a history of myocardial infarction were excluded in the ENESTnd study [27].

Regardless of the variations across the different TKI studies, it is important to consider that both nilotinib and ponatinib have been associated with development of vascular ischemic events over time [7, 28]. In the 5-year follow-up to the ENESTnd study, a cumulative increase in the frequency of cardiovascular events was observed in nilotinib-treated patients [9]; furthermore, the FAERS analysis reported a unique association of peripheral and cardiac vascular events with nilotinib [8]. Response to ponatinib was investigated in patients with CML previously treated with dasatinib or nilotinib or who had a BCR-ABL1 T315I mutation, in the PACE study (*n* = 449) [29]. The proportion of patients with vascular ischemic events on ponatinib increased from 9 to 17% from 11 to 24 months of follow-up [7]. While higher doses of nilotinib or ponatinib have been associated with increased vascular ischemic events [6], neither dasatinib dose nor disease phase affected the frequency of cardiovascular ischemic events in this analysis. The varied effect that the different BCR-ABL1 TKIs have on development of cardiovascular AEs should be considered in future studies as an opportunity to examine physiologic targets of TKIs to gain a better understanding of arterial ischemic events in general.

The relation between risk factors for experiencing a cardiovascular ischemic event (preexisting ischemic heart disease and non-cardiac atherosclerosis, smoking, hypertension, diabetes, and hypercholesterolemia) and event incidence was also investigated in this analysis. Approximately 80% of the cardiovascular ischemic events reported in patients receiving dasatinib occurred in patients who had a history of and/or risk factors for atherosclerosis. The ENESTnd study also reported a higher incidence of cardiovascular events in nilotinib-treated patients with high versus low cardiovascular risk scores [9]. Furthermore, the likelihood of experiencing an arterial thrombotic event was reported to be associated with cardiovascular risk in ponatinib-treated patients in the PACE trial [30]. Aging is also associated with an increased incidence of cardiovascular disease, as 51% of US adults with cardiovascular disease are aged ≥60 years [31]. In an observational study of patients with CML-CP treated with first-line imatinib, dasatinib, or nilotinib in the community setting (SIMPLICITY; NCT01244750), cardiovascular event-related hospitalizations were higher in older patients [32].

In this analysis, the percentage of patients with a cardiovascular ischemic event, when treated with dasatinib, in the pooled Ph+ population and DASISION increased with age. Despite the increased age of patients from READY, the rate of events in both arms of this study did not exceed the rates in the studies of patients with Ph+ leukemia or what was expected when compared to age- and sex-adjusted cohorts used for the SIRs. The reasons are unclear why there is not a more defined relationship between occurrence of cardiovascular ischemic events and age in READY. The READY study did not include a population of patients who were <44 years of age (31 and 47% of the pooled and DASISION populations, respectively), which could possibly make identifying an association between events and age difficult. The median daily dose of dasatinib for patients in READY was 99 mg, which is comparable to the medians of 88 mg received by patients with CML-CP and 125 mg received by patients with CML-AP/BP or Ph+ ALL in the pooled population; this suggests that dosage differences do not account for the lack of age-associated cardiovascular events in patients from READY. One possibility is that the presence of concomitant medications, such as docetaxel and/or prednisone, may have altered the risk. Another explanation may be the previous androgen deprivation therapy (ADT) received by the READY population. There is evidence that ADT may be associated with cardiotoxicity and that related AEs are more common in patients with preexisting cardiovascular comorbidities [33]. Therefore, if the risk of experiencing a cardiovascular event was already increased in the READY population due to ADT, any effect of age on these events in patients who did or did not receive dasatinib may be more difficult to observe.

There are limitations to the SIR analysis owing to differences in data collection between international clinical trials and US insurance claims populations. Some cardiovascular ischemic events identified in clinical trials were not identified in the MarketScan database, potentially because the terminology may not be equivalent to what was reported in clinical trials. Also, reporting of AEs in the MarketScan database was voluntary, so the incidence of cardiovascular ischemic events is most likely underestimated. The clinical trial populations also differed from the general population due to inclusion/exclusion criteria, concomitant medications, performance status, and organ function of patients based on clinical trial eligibility criteria, which cannot be controlled for in the external reference populations.

In summary, in this analysis of a large population of patients who received dasatinib across a clinical trial program, a low proportion of patients experienced cardiovascular ischemic events across all of the doses tested. Moreover, the rates of cardiovascular ischemic events were not higher than expected when compared with CML or prostate cancer populations from publicly available databases in the USA, after adjusting for age and sex. Among patients who experienced a cardiovascular ischemic event, the majority had a history of prior arterial ischemic events or risk factors for atherosclerosis. In contrast to what has been observed with other TKIs, events were largely restricted to within the first year after initiation of dasatinib, with no evidence of a cumulative risk. Physicians should carefully assess preexisting comorbidities and risk factors when selecting treatment for patients with Ph+ leukemia and should monitor patients closely during therapy.

## Electronic supplementary material

ESM 1 (DOC 46 kb).
