# Supplementary material for: Evaluation of cardiovascular ischemic event rates in dasatinib-treated patients using standardized incidence ratios
Source: Ann Hematol. 2017 May 22;96(8):1303–13. doi: 10.1007/s00277-017-3012-z (PMC5486782; doi:10.1007/s00277-017-3012-z)
Supplement: Supplementary file 1 — (DOC 46 kb). [file 277_2017_3012_MOESM1_ESM.doc]

**Evaluation of cardiovascular ischemic event rates in dasatinib-treated patients using standardized incidence ratios**

**Online Resource**

**Journal:** *Annals of Hematology*

Giuseppe Saglio • Philipp le Coutre • Jorge Cortes • Jiří Mayer • Philip Rowlings • François-Xavier Mahon • Glenn Kroog • Kyna Gooden • Milayna Subar • Neil P. Shah

Correspondence: Giuseppe Saglio, MD

Clinical and Biological Sciences of the University of Turin

San Luigi Hospital

10043 Orbassano-Torino, Italy

Tel: + 39 3356061708

Fax: + 39 0119038636

E-mail: giuseppe.saglio@unito.it

**Online Resource Methods**

**Patient populations**

**Online Resource Table S1** Trial and disease state information for study populations

| Trial | Study population | *N* |
| --- | --- | --- |
| Pooled population |  | 2712 |
| NCT00103701 (002)^a^ | CML or Ph+ ALL, R/I to imatinib | 91 |
| NCT00101647 (START-A)^b^ | CML-AP, R/I to imatinib | 174 |
| NCT00101816 (START-B)^b^ | CML-MBP, R/I to imatinib | 109 |
| NCT00101660 (START-C)^b^ | CML-CP, R/I to imatinib | 387 |
| NCT00101595 (START-L)^b^ | CML-LBP or Ph+ ALL, R/I to imatinib | 94 |
| NCT00103844 (START-R)^b^ | CML-CP, R to imatinib | 141 |
| NCT00123474 (034)^c^ | CML-CP, R/I to imatinib | 662 |
| NCT00123487 (035) | CML-AP/BP or Ph+ ALL, R/I to imatinib | 609 |
| NCT00481247 (DASISION)^d^ | CML-CP, newly diagnosed | 258 |
| NCT00529763 (160) | CML or Ph+ ALL, R/I to imatinib | 121 |
| NCT01357655 (363) | CML-CP, newly diagnosed | 66 |
| DASISION |  | 519 |
| NCT00481247 (DASISION) | CML-CP, newly diagnosed,  treated with dasatinib | 259 |
|  | CML-CP, newly diagnosed,  treated with imatinib | 260 |
| READY |  | 1522 |
| NCT00744497 (227) | Prostate cancer, treated with docetaxel/prednisone plus dasatinib | 762 |
|  | Prostate cancer, treated with docetaxel/prednisone plus placebo | 760 |
| *ALL* acute lymphoblastic leukemia, *AP* accelerated phase, *BP* blast phase, *CML* chronic myeloid leukemia, *CP* chronic phase, *I* intolerant to imatinib, *LBP* lymphoid blast phase, *MBP* myeloid blast phase, *R* resistant to imatinib  ^a^Some patients crossed over to NCT00978731 (039) for long-term follow-up  ^b^Some patients in these trials and 039 crossed over to NCT00982488 (188) for long-term follow-up  ^c^Analysis based on 7-year minimum follow-up  ^d^Analysis based on 5-year minimum follow-up | | |

**External reference populations**

MarketScan® (Truven Health Analytics, Ann Arbor, MI, USA) consists of data from commercial health plans, Medicaid, Medicare, and self-insurance for several million individuals across the United States. Three external reference populations were identified from MarketScan using SÆfetyworks (United Biosource Corporation, Blue Bell, PA, USA), a software application designed to support pharmacovigilance processes by analyzing associations between drug, condition, procedure, and hospitalization occurrences within large observational databases, such as electronic health records and insurance claims databases.

Patients eligible for inclusion in the chronic myeloid leukemia or prostate cancer populations had two or more diagnostic codes for chronic myeloid leukemia/prostate cancer, had no prior dasatinib use, were aged ≥18 years at the index date, and had been enrolled in the database for ≥180 days before the index date. Patients included in the general population were aged ≥18 years, had been enrolled in the database for ≥180 days before the index date, and had no codes compatible with cardiovascular ischemic events prior to the follow-up start date. The index date was the first date that patients met all eligibility requirements.

**Analysis**

Medical Dictionary for Regulatory Activities preferred terms included acute coronary syndrome, acute myocardial infarction, angina pectoris, angina unstable, arteriosclerosis coronary artery, cardiac discomfort, coronary arterial stent insertion, coronary artery disease, coronary artery occlusion, coronary artery stenosis, electrocardiogram (ECG) signs of myocardial ischemia, ECG T-wave abnormal, ECG T-wave inversion, myocardial infarction, myocardial ischemia, silent myocardial infarction, troponin I, troponin I increased, and troponin increased. Preexisting risk of cardiovascular ischemic events was evaluated in the clinical trial patient subsets with and without a cardiovascular ischemic event on the basis of risk factors provided at enrollment, including hypertension, smoking, diabetes mellitus, hypercholesterolemia, and preexisting vascular disease (ischemic heart disease and noncardiac atherosclerosis). Across all of the dasatinib trials, adverse events were reported if they occurred any time after the first dose or within 30 days of last study dose.
